# Mineral Waters—Their Medicinal and Hygienic Advantages

**Published:** 1883-05-20

**Authors:** T. S. Powell


### MINERAL WATERS—THEIR MEDICINAL AND HYGIENIC ADVANTAGES.

It would be difficult to believe that the dullest intellect of man could look abroad upon the universe and not be profoundly impressed with the wonderful beneficence of the Creator, as shown in the beauty, utility, and grandeur of nature; but, in no case is this immeasurable love and goodness of the Divine Giver more manifest to the mind than when we learn of the varied and delightful resources for the healing of disease as found in their primeval purity in so many parts of the world.

The medical scientist, after long years of successful experiment, can discover remedies that may arrest the progress of disease, but no chemical art can presume to rival the exactness and completeness of nature's laboratory, in which are wrought remedial agents fresh from the gracious and omnipotent hand of the Great Physician!

A doctor, learned in medical lore, may safely prescribe for his patient a certain quantity of pills and potions, but he cannot compound the healing waters that gush cold, or hot and clear, from the rocky hill-side, nor the refreshing mountain br ezes that kiss into bloom the invalid's pallid cheek; neither can he paint the wonderful panorama of nature's most beautiful and sublime attributes—the views of which cause the weary eye of the sick and languid sufferer to brighten with a new interest in life; that make his pulses quicken, and the blood bound more buoyantly and healthfully through his sluggish veins. These are the delightful and efficacious hygienic auxiliaries to health that a beneficent Creator furnishes to aid so materially the sick in regaining physical strength and vigor. At no place in this country can the seeker of health and wholesome recreation more successfully find these assistants than at the Blue Ridge Springs, of Botetourt county, Virginia. The great healing qualities of these waters were not known to the public until within the last few years, but the delightful temperature, grand mountain scenery, pure air, and the wonderful virtues of the springs are fast bringing this summer resort into favorable notice as a place for health, comfort and pleasant recreation. Though 1,300 feet above the level of the sea, this charming retreat has its elegant and commodious hotel immediately on the line of the Norfolk & Western railroad, so that the visitor, greatly to his gratification, has none of the annoyances and discomfort of hack-driving, over rough roads, or of transfer to a hotel omnibus, but, at a few steps from the cars, finds himself greeted by his smiling and courteous landlord, Mr. Phil F. Brown, and is immediately consigned to a cool and restful chamber, with all the comfortable and elegant resources of the house and ground at his command.

The springs are within a few steps of the hotel, and from the rear of the hotel a long and picturesque bridge crosses a lovely stream of water, and reaches from the spacious and beautiful lawn of the hotel with its terraced cliff, to that upon the other side of the stream; and here, upon this lofty summit of the Blue Ridge, surrounded by grand peaks that pierce the bending blue skies, lie, spread out before the beholder, scenes of such sublimity and exalted beauty as one could not imagine from a mere passing glance on the railway in front of the hotel.

A number of commodious and comfortable cottages, in the near vicinity of the Springs, are connected by the handsome covered bridge, referred to, with the hotel, so that invalids, or families with small children, can have their choice of a retreat as private and pleasant as could be desired.

The grounds about the hotel, with flowers, fountains and beautifully shaded promenades, are extensive and lovely, and there is a miniature lake with pleasure boats, also a choice vineyard, and thousands of fine fruit trees, the product of which regales the guests of "mine host," who is ever on the alert for their comfort and pleasure.

A ball-room, with good music, an excellent ten-pin alley, and a billiard saloon, afford pastime for a variety of tastes among visitors; and to those who wish to visit places of interest in the vicinity, the Blue Ridge Springs is a most convenient point from which to make excursions to the Luray Caverns, Natural Bridge of Virginia, and the Peaks of Otter.

A great iron mining interest has recently been developed in the immediate vicinity of these Springs, and two railroads have been built from the main line of the Norfolk & Western out to the base of the mountain. Near the terminus of the road has been found a beautiful cascade of pure mountain water, affording a charming spot for bathing.

Comparatively few persons of even more than ordinary intelligence are fully aware that *inertia is death* to animated matter; that invalids, with the best medical advice, if they would regain health must have surroundings that will lift them "out of themselves," as it were; that will interest them, pleasantly excite their emotional and intellectual natures, make their pulses quicken, and answer, with a responsive thrill, to the beauty and cheerful influence of the sights and sounds about them, and with which they come in daily contact. With all these healthful assistants and delightful recreations, the Blue Ridge Springs are abundantly provided.

To the more impressible and highly wrought nervous constitution of females, the advantages above referred to are eminently suited. These advantages are found not alone in the peculiar position and surroundings of this magnificent sanitarium, but the analysis of the water unmistakably indicates their adaptation to a great variety of affections. This analysis may be seen in the advertisement of the proprietor, from which it appears that the water is of the saline class, holding in solution a happy proportion of neutral salts, so arranged in nature's alembic as to constitute laxative, purgative, diuretic, tonic, and alterative properties, according to the quantity used.

With a scientific and judicious physician to direct its use in any particular case, the water of these springs may be made available for the successful treatment of a great variety of conditions. Indeed, with the proper use of the water, conjoined with the pure and wonderfully invigorating atmosphere of this favored locality, we can scarcely imagine any disease at all amenable to treatment, that could fail of benefit or relief from these waters. In obstinate chronic diseases of both sexes, especially in uterine affections and derangements requiring local or general treatment, failures often occur in private practice by reason of unfavorable surroundings and depressing influences at home. But with the advantages of a superior watering place, and suitable food and dietetic caution, far better results may be obtained from local treatment, because the physician has the great advantage of favorable surroundings and medicinal waters which constitute powerful auxiliaries in the treatment of this class of diseases.

Indeed, it is conceded by every intelligent physician, and is abundantly verified in the experience of thousands, that diseased conditions of every kind can be far more successfully treated at a good watering place, such as we have described, than could possibly be expected under other and less favored conditions. Hence it is that the judicious physician often advises his patients to visit these favored resorts, where merely functional diseases usually recover rapidly, and where organic and obstinate chronic affections are temporarily benefitted. and we doubt not could often be permanently cured, if placed in charge of a skillful resident physician, who, by the aid of modern scientific means and appliances, the waters, and favorable conditions of the place, would be able to accomplish results which, under other circumstances, he could not possibly do.

Such have always been my views in regard to medicinal Springs, and the proper methods of using their waters. The physician at home is not able to supply and bring to bear these great advantages and facilities, which nature, in her bounty and wisdom, has supplied for the benefit of suffering humanity. When these medicinal watering places are extensively and beautifully improved, and supplied with bathing arrangements, hot and cold, and all the modern facilities, they will constitute the best places for the restoration of health and the treatment of chronic diseases, especially those peculiar to females; and there is at this locality what appears a special provision for some conditions, in the fact that in addition to the principal waters, to the analysis of which we have referred, there is a Chalybeate Spring, strong in iron, which may be secondarily used in a certain class of cases, which have been prepared for its use by other waters or medical treatment. In some instances it is primarily demanded. Particularly is this true of cases of low anæmic character, attended with atony, or great debility, and unattended with irritation, or an inflammatory state or tendency in any organ.

In this fact, the Blue Ridge Springs possesses peculiar and unusual advantages as a sanitarium for the treatment of almost all diseases.

For variety and excellence of her health giving waters, for magnificent scenery and salubrious atmosphere, Virginia has been especially favored by a beneficent Creator. She has been called the Mother of States, then let her afflicted sons and daughters, from every State, come and partake of her bounties, enjoy her generous hospitality, breathe her mountain air, and drink of her healing waters, all of which she so bounteously offers to her children throughout this heaven-favored land of ours.

T. S. POWELL.

